# Peripheral Blood Smear Findings in COVID-19

**DOI:** 10.4274/tjh.galenos.2020.2020.0262

**Published:** 2020-11-19

**Authors:** Maryame Ahnach, Fadwa Ousti, Sara Nejjari, Mouad Sqalli Houssaini, Nouzha Dini

**Affiliations:** 1 Cheikh Khalifa International University Hospital, Mohammed VI University of Health Sciences, Department of Hematology, Casablanca, Morocco; 2 Mohammed VI University of Health Sciences, National Reference Laboratory, Casablanca, Morocco; 3 Mohammed VI University of Health Sciences, Cheikh Khalifa International University Hospital, Casablanca, Morocco

**Keywords:** COVID-19, Morphology cells, Blood smears

## To the Editor,

In December 2019, coronavirus disease 2019 (COVID-19) emerged in Wuhan, China, and caused a global pandemic. The disease has a systemic manifestation including the hematopoietic system. It is clear that the hematologic laboratory will play an essential role in this crisis, contributing to patient screening, diagnosis, and prognosis. To date, complete blood counts (CBCs) have been recommended as useful tools for patient monitoring. Quantitative hematologic abnormalities have been reported since the first published papers and all blood cells can be affected during COVID-19, mainly leukocytes and platelets [1]. Blood smears are not performed systematically, but due to the presence of quantitative anomalies, a qualitative evaluation is useful for analyzing morphological changes during COVID-19.

**Figure 1:**
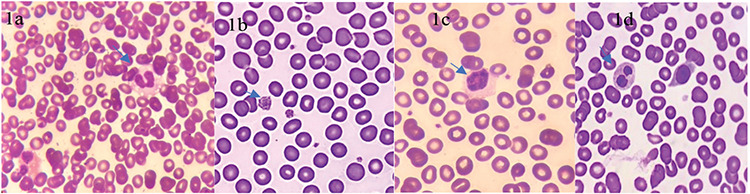
Blood smears of patients with COVID-19 (May-Grunwald-Giemsa): a) eosinophil containing multiple vacuoles; b) giant platelets with different sizes; c) circulation of a large lymphocyte; d) neutrophil granulocyte with marked hypogranular cytoplasm and hyposegmented nucleus.

In Morocco, with a record of 6741 confirmed cases and 192 deaths, the situation is less critical than in Europe and the United States [2]. A COVID unit was created in our hospital to provide assistance and care to patients from the Casablanca region. We have had up to 146 patients with COVID-19 as of May 16, 2020. The CBC parameters of our patient (n=146) showed neutropenia (7.5%), hyperleukocytosis (8%), eosinopenia (47%), monocytosis (9.5%), lymphopenia (46%), and thrombopenia (10%). We evaluated 15 first peripheral blood smears at admission and we found morphological abnormalities concerning leukocyte and platelet lineages. By observing blood smears colored by May-Grunwald-Giemsa stain, we noted the characteristics of a neutrophil granulocyte with dysmorphic morphology marked by hypogranular cytoplasm and hyposegmented nucleus. We observed the presence of atypical eosinophils containing multiple vacuoles. Rare activated lymphocytes and large monocytes were found in some peripheral blood films. Platelet morphology also showed frequent anomalies, mainly consisting of giant platelets with different sizes (Figure 1).

Very few articles have been published on blood morphology during COVID-19 infection. Two recent publications described the blood smears of infected patients, where the morphology of the neutrophil and platelet lineages showed very frequent anomalies in terms of nuclear morphology, cytoplasmic granulation, and the presence of atypical and immature cells [3,4]. Like most viruses that impact hematopoiesis and the immune system during developmental stages [5], COVID-19 causes blood cells to change by inflammatory mechanisms and the perturbation of the myelopoiesis system [6]. Moreover, it appears to have more serious effects, with deep cytopenia predictive of the severity [7]. CBCs completed with peripheral blood smears can detect the impact of the virus on the blood, reflecting early inflammatory signs.

Our preliminary results remain limited and more investigation is required to study the reversibility of these abnormalities and their impact on severity. Compared to all inflammatory biomarkers, observation of blood cells can be a simple alternative for the first triage and early identification of the infection.
